# Recent Trends and Advancements in CRISPR-Based Tools for Enhancing Resistance against Plant Pathogens

**DOI:** 10.3390/plants12091911

**Published:** 2023-05-08

**Authors:** Munazza Ijaz, Fahad Khan, Haitham E. M. Zaki, Muhammad Munem Khan, Khlode S. A. Radwan, Yugen Jiang, Jiahui Qian, Temoor Ahmed, Muhammad Shafiq Shahid, Jinyan Luo, Bin Li

**Affiliations:** 1 State Key Laboratory of Rice Biology and Breeding, Ministry of Agriculture Key Laboratory of Molecular Biology of Crop Pathogens and Insects, Institute of Biotechnology, Zhejiang University, Hangzhou 310058, China; 2 Tasmanian Institute of Agriculture, University of Tasmania, Prospect, TAS 7250, Australia; 3 Horticulture Department, Faculty of Agriculture, Minia University, El-Minia 61517, Egypt; 4 Applied Biotechnology Department, University of Technology and Applied Sciences-Sur, Sur 411, Oman; 5 Department of Plant Breeding and Genetics, University of Agriculture, Faisalabad 38000, Pakistan; 6 Plant Pathology Department, Faculty of Agriculture, Minia University, El-Minia 61517, Egypt; 7 Agricultural Technology Extension Center of Fuyang District, Hangzhou 311400, China; 8 Department of Plant Sciences, College of Agricultural and Marine Sciences, Sultan Qaboos University, Al-Khod 123, Oman; 9 Department of Plant Quarantine, Shanghai Extension and Service Center of Agriculture Technology, Shanghai 201103, China

**Keywords:** phytopathology, CRISPR, resistance, genome editing

## Abstract

Targeted genome editing technologies are becoming the most important and widely used genetic tools in studies of phytopathology. The “clustered regularly interspaced short palindromic repeats (CRISPR)” and its accompanying proteins (Cas) have been first identified as a natural system associated with the adaptive immunity of prokaryotes that have been successfully used in various genome-editing techniques because of its flexibility, simplicity, and high efficiency in recent years. In this review, we have provided a general idea about different CRISPR/Cas systems and their uses in phytopathology. This review focuses on the benefits of knock-down technologies for targeting important genes involved in the susceptibility and gaining resistance against viral, bacterial, and fungal pathogens by targeting the negative regulators of defense pathways of hosts in crop plants via different CRISPR/Cas systems. Moreover, the possible strategies to employ CRISPR/Cas system for improving pathogen resistance in plants and studying plant–pathogen interactions have been discussed.

## 1. Introduction

**Figure 1:**
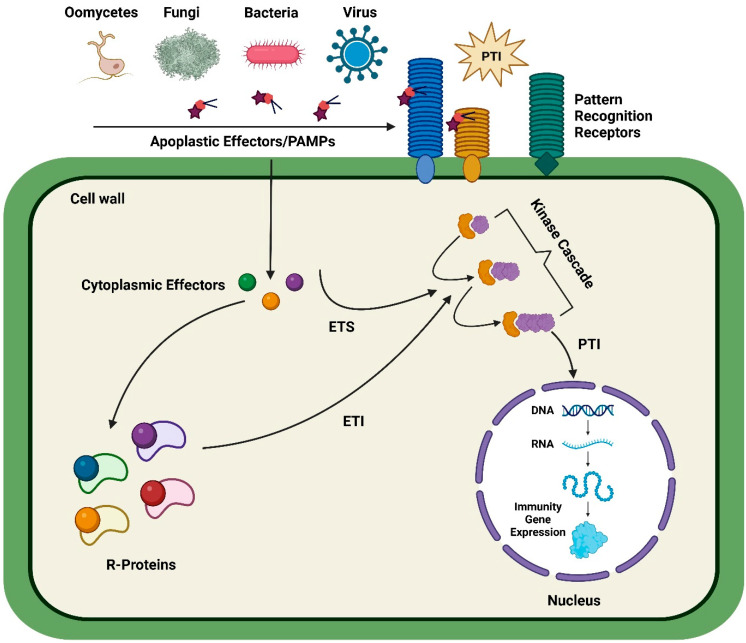
An illustration of the plant defense system. In the diagram, it is illustrated how plant immunity (PTI and ETI), resistance to pathogens induced by ETS, and genes involved in the interaction between plants and pathogens are intricately related. The PAMPs (protein-based antimicrobial peptides) in the extracellular environment are recognized by plasma membrane-localized receptors (PRR) from a variety of pathogen types such as viruses, bacteria, or fungi, and the response is triggered by either the PAMP-triggered immunity (PTI) or the Effector-triggered immunity (ETI). Pathogen effectors are regulatory molecules that modify host proteins to establish their growth and initiate the process of effector-triggered susceptibility (ETS). The kinase cascade also plays a role in the crosstalk between PTI and ETI pathways and in the regulation of systemic acquired resistance (SAR).

The world’s population is increasing day by day and is expected to exceed 9.8 billion in 2050, so an excessive amount of food production is expected to fulfill the dietary needs of the growing population [1,2]. Global food security is at risk due to the ominous presence of plant pathogens, including bacteria, viruses, fungi, and parasites [3,4]. Therefore, it is necessary to develop resistance in plants to increase the production of crops to meet the global food demand [5]. It is a well-known fact that plants and pathogens are in a constant war to develop immunity against each other [6,7]. The plants have developed “pattern triggered immunity (PTI)” and “effector triggered immunity (ETI)” to avoid pathogens [8]. In general, “pathogen associated molecular patterns (PAMPs)” rapidly activates the PTI via “pattern recognition receptors (PRRs)” [9,10]. These basic responses of plants, including the callose deposition, generation of “reactive oxygen species (ROS)”, and transcriptional reprogramming, prevent the spread of weak pathogens in plants [11]. For fighting with the first line of defense, effectors are secreted by pathogens to overcome PTI and modify the physiology of host cells, which results in the “effector-triggered susceptibility (ETS)” [12]. However, the intracellular immune system of pathogen-resistant plants can recognize the effector proteins and initiates ETI, which usually leads to localized cell death in the plant to reduce the spread of insect pests [13]. However, some of the virulent pathogens can modify the ETI-stimulating effectors and can overcome the host ETI response [14,15]. Therefore, an endless fight remains continued between the two defense systems of host plants and pathogens [16]. A schematic representation of the plant defense mechanism is illustrated in Figure 1.

The homing endonucleases have revolutionized genome-editing (GE) studies with the advent of techniques such as “zinc-finger nuclease (ZFNs)”, transcription activator-like effector nucleases (TALENs), and CRISPR/Cas systems that can precisely induce mutations in DNA and have transformed the genetic modification techniques of plants in recent years [17,18]. The CRISPR/Cas9 system was primarily found in bacteria as an RNA-mediated adaptive-immune system activated in response to different viral infections [19,20,21]. Nowadays, CRISPR-based tools have become an alternative to TALENs and ZFNs, due to their high efficiency and simplicity in genome engineering technologies [22]. From CRISPR-Cas9 and CRISPR-Cpf1 to CRISPR-assisted gene activation, epigenetic modulation, RNA-guided RNA degradation, and synthetic biology, researchers have explored a variety of approaches to enhance plant resistance [23]. The CRISPR-based genome-editing technologies have been widely adapted to study plant–pathogen interactions that include host defense response against viruses, bacteria, fungi, and other pathogens [24,25]. In the realm of epigenetics, modifying the histone code in plants through CRISPR-based tools has been explored as a means of enhancing plant resistance [26,27]. By modulating the chromatin accessibility of the genomic DNA, researchers have successfully augmented the transcriptional output of defense-related genes, thereby fortifying the plant’s innate immunity against various pathogenic agents [28,29]. The improvement of crop resistance to pathogens and identification of defense-related genes to ensure sustainable agriculture development and food safety have become possible with the CRISPR technology [30]. In this review, we have delved into the recent advancements and trends in CRISPR-based tools for boosting plant resistance, including a comprehensive overview of the current state of the field and the most promising avenues for future research. We have also reviewed distinct characteristics of CRISPR-based targeted mutagenesis technologies, as well as discussed their current uses for studying plant–pathogen interactions. Additionally, we have discussed the possible limitations and future directions of CRISPR/Cas9 systems in genetic modifications related to plant protection and development.

## 2. Different CRISPR-Based Tools

The CRISPR/Cas systems have been classified on the bases of structural, functional, and phylogenetic characteristics of Cas proteins into two main classes that are “Class 1 (types I, III and IV)” and “Class 2 (types II, V and VI)”. These different CRISPR/Cas systems have different mechanisms for the target interference and biogenesis of guide RNA [31]. The CRISPR-based Class 2 endonucleases have been broadly used for manipulating nucleic acid sequences [32]. Type-II CRISPR/SpCas9 has been widely used and best characterized for targeted genome editing in plants [33]. This system has been originated from *Streptococcus pyogenes* and is composed of 3 major elements (a small mature “CRISPR RNA (crRNA)”, “SpCas9 nuclease”, and “trans-activating small RNA (tracrRNA)”) [34,35]. However, the modern and updated CRISPR system has only two components guide RNA (gRNA, which is made by artificial fusion of both crRNA and tracrRNA) and SpCas9 nuclease [36].

The SpCas9 nuclease reaches the targeted site of DNA with the help of gRNA that has 17–20 complementary nucleotides (nt) which recognize a “protospacer adjacent motif (PAM) sequence”, 5′-NGG-3′ [37]. The selection of target sequences is mainly restricted due to the need for the specific PAM sequence [38]. Two major approaches have been developed to overcome this restriction: (a) the variations of Cas9 protein to identify various PAM regions and (b) the use of different Cas proteins obtained from different organisms [39]. The modified or engineered xCas9, SpCas9-NG, SpRY, and SpG that recognize the 5′-NG-3′ PAM sequence can increase the efficiency of SpCas9 mediated genome editing [40,41]. Variations of SpCas9 that can recognize a variety of PAM sequences have been successfully applied to plants for genome editing. Meanwhile, the off-target mutations can probably impact phenotype, but they could be eradicated via back-crossing and genetic segregation in plants [42]. To reduce the off-target mutations, the highly specified variants of SpCas9 have been engineered and used for base editing and targeted mutagenesis in plant species [43]. Interestingly, the PAM sequence, 5′-NNG-3′, is recognized by an orthologous Cas9 protein obtained from *Streptococcus canis* (scCas9).

The ScCas9 system has been effectively employed for base editing and targeted mutagenesis in *Oryza sativa* [44]. A mutant of ScCas9 known as ScCas9++ can also be used as a useful genome editing tool in different plant species. The Cas12a and CRISPR-based complex consisting of Cas3d/Cas5d/Cas6d/Cas7d/Cas10d have also been used for genome editing of plants. In Arabidopsis, the targeted mutagenesis has been achieved by applying a temperature-sensitive and highly resistant Cas12a protein and its variants ttLbCas12a, LbCas12a, and enLbCas12a [45]. Examples of Cas nucleases, their PAM sequences, and the genetically modified crops have been listed in Table 1. Many changes in important agronomic traits, such as yield and pathogen resistance, are due to “single nucleotide polymorphisms (SNPs)”. So, many precise base-editing tools such as “adenine base-editor (ADE)” and “cytosine base-editor (CBE)” have been made for inducing nucleotide substitutions in different crops. These base editing tools have been engineered by either adenine deaminase or cytosine deaminase to genetically impair Cas9, which can induce adenine to guanine and cytosine to thymine substitutions at target sites, respectively [46].

## 3. CRISPR-Based Genome Editing of Plants for Disease Resistance against Pathogens

Newly emerged CRISPR-based genome-editing technologies play a vital role in developing disease resistance against bacteria, fungi, viruses, and other pathogens. In the below section, we have discussed some recent trends in the disease resistance achieved by CRISPR/Cas system in different pathogens.

### 3.1. Disease Resistance against Bacteria

The type III effectors are generally secreted in plants during bacterial infections, which interrupt the defense of host cells and activate susceptibility (S) genes for the development of disease. Editing S genes using CRISPR-Cas technology can reduce the susceptibility of plants to bacterial diseases without compromising plant growth or yield. By precisely targeting and modifying specific S genes, it is possible to reduce the ability of *Xanthomonas* to colonize plants and cause disease. One promising approach is to edit the susceptibility genes (S genes) in crop plants. S genes play an essential role in the plant–microbe interaction, as they encode proteins that are required for bacterial colonization and disease development. For example, the SWEET gene family encodes sugar transporters that are essential for bacterial pathogenesis. Similarly, the LOB1 gene is involved in regulating plant immunity and is required for Xanthomonas to cause disease in certain crops [61]. This approach has already been successfully demonstrated in a variety of crops, including rice, tomato, and citrus. CRISPR-Cas editing works by introducing specific changes into the DNA sequence of the target gene. This technology uses a programmable RNA molecule that guides a nuclease enzyme to the target site in the genome, where it creates a double-stranded break [62]. The cell’s repair mechanisms then fix the break by either deleting or inserting new DNA bases, resulting in a modified gene sequence. Editing S genes using CRISPR-Cas technology is a promising approach for controlling bacterial diseases in crops. By targeting and modifying specific genes that are essential for bacterial colonization and disease development, it is possible to reduce the susceptibility of plants to *Xanthomonas* and other bacterial pathogens. This approach could reduce the reliance on agrochemicals and lead to more sustainable and effective disease control strategies for farmers [63]. Hence, CRISPR/Cas system targets the negative regulators of innate immunity and S genes to improve plant resistance against” bacteria [61]. For instance, the resistance against “*Xanthomonas oryzae* pv. *oryzae (Xoo)”* can be achieved by knocking out the Os8N3 or OsSWEET11 gene of rice that is the target for TALEs [62]. Strong resistance against many Xoo strains can be conferred by targeting the “TALE-binding elements (EBEs)” present in the promoter sequences of the “OsSWEET11” and “OsSWEET14” genes [63]. Furthermore, broad-spectrum resistance has been induced when the promoter sequence of OsSWEET11/OsSWEET13/OsSWEET14 was targeted by CRISPR/Cas system [64]. The “CRISPR/Cas12” and “CRISPR/SpCas9” tools have also been proven beneficial for inducing resistance against many plant pathogens. For instance, resistance against citrus canker disease that is caused by *Xanthomonas citri* pv. *citri* can be achieved by targeting the promoter sequence of the CsLOB1 gene in citrus [65]. Moreover, the CRISPR/spCas9 system has generated germplasm of tomato plants that is resistant to “*Pseudomonas syringae* pv. *tomato DC3000*” [66].

### 3.2. Disease Resistance against Fungi

The fungal pathogens start host colonization which is a quite complex process [67]. The negative regulators and S genes are major targets of CRISPR/Cas systems to improve tolerance against fungi in crops [68]. For instance, a homolog of a well-known S gene known as mildew resistance locus O (MLO) in wheat (TaMLOs) was knock out by the CRISPR/SpCas9 system to confer tolerance against *Blumeria graminis* f.sp. *tritici* (a fungal pathogen) that is responsible for powdery mildew disease [69]. Moreover, the resistance to powdery mildew fungus of tomato *Oidium neolycopersici* was conferred by knocking out its homolog (SIMLO1) in tomato [70]. Similarly, in wheat, the resistance against powdery mildew was achieved by knocking out three copies of TaEDR genes simultaneously [71]. One more important case is the “OsERF922 gene” which is responsible for encoding a TF (transcription factor) belonging to the family of “ethylene response factor” in *Oryza sativa* and has a significant function in positive and negative regulations of defense mechanism. The editing of OsERF922 by the CRISPR/SpCas9 system has enhanced the tolerance to “blast fungus (*M. oryzae*)” in rice without impacting the growth and other important agronomic traits of plants [72]. The tolerance against *M. oryzae* has been enhanced by switching off the BSR-K1 gene with the help of the CRISPR/SpCas9 system in rice [73]. The CRISPR/Cas9 system can also repair defective R genes through precise base editing for improving the tolerance against diseases in crops. For instance, in the recessive allele of Pi-d2, an SNP at position no. 441 has been found to be linked with achieving tolerance against *M. oryzae* [40]. Compared to the conventional breeding method, the way of precise base editing is faster and more efficient for enhancing disease resistance in crops.

### 3.3. Diseases Resistance against Oomycetes

The oomycetes are fungus-like eukaryotes that belong to the family Chromista [74]. The *Pythium* spp., *Phytophthora* spp., and *Peronospora* spp. are the most deleterious pathogenic oomycetes [75]. The studies mitigating the risk of oomycetes disease via CRISPR-based technologies are limited and mainly focus on editing the effectors [76]. Targeting the transient expression of TcNPR3 in the leaves of Theobroma coca has achieved resistance against the pathogen *P. tropicalis* [77]. It is well known that NPR3/NPR4 function as co-repressors of transcription during the salicylic acid-responsive defense mechanism in Arabidopsis, so it is needed to modify their homologs in other economically important crops to confer resistance against oomycetes species [77,78].

### 3.4. Disease Resistance against Plant Viruses

The genome-editing tools have evolved quite fast in recent years and CRISPR/Cas systems have been proven as highly effective platforms to engineer virus resistance [79]. Resistance against viruses can be attained either by modifying the host elements that render the replication of viral species or by directly destroying and targeting various viral genomes to stop their replication [80]. The cap-binding protein or the eukaryotic initiation factor 4E (eIF4E) plays a key role in enhancing plant virus susceptibility [81]. The inactivation of eIF4E has been found to develop innate immunity in several plant species against the potyviruses (a family of viruses called potyviridae). A study has shown the role of eIF4E modification in cucumber plants via CRISPR/SpCas9 resulted in the development of resistance against several viruses, including “*papaya ringspot mosaic virus-W (PRSMV-W)*”, “*zucchini yellow mosaic virus (ZYMV)*”, and “cucumber vein yellowing virus (CVYV)” [82]. A CRISPR/SpCas9 mediated mutagenesis of eIF4E in A. thaliana and cassava led to the reduction in the symptoms of *turnip mosaic virus (TuMV*) infection and cassava “*brown streak disease*” [83,84]. Tolerance against rice “*tungro spherical virus (RTSV)*” was found when eIF4G was targeted for modification through CRISPR/SpCas9 system. The cytidine base editing in *A. thaliana* involving a C > G conversion at N176K in eIF4E1 (wild type) led to achieving tolerance against the “clover yellow vein virus” [85,86].

The DNA viruses with a single-stranded circular genome, also known as the Geminiviruses, can cause severe harm to economically valuable crops such as sugar beet, tomato, and pepper. Several studies have directly targeted the genome of Gemini viruses using CRISPR/SpCas9 [87]. The CRISPR/SpCas9 system targeting the intergenic region and replication-associated protein (Rep protein) was used to target the genome of “bean yellow dwarf virus (BeYDV)” and “*beet severe curly top virus (BSCTV)*” [84,88]. The modified viruses were then transformed into *A. thaliana* and *N. benthamiana*. An enhanced level of tolerance was observed in the transformed plants [89]. Similarly, viral resistance against tomato yellow leaf curl virus (TYLCV) was conferred using SpCas9 and sgRNA targeting the Rep protein and coat protein in a tomato plant and *N. benthamiana* [90]. Other studies had depicted the enhancement of tolerance to *banana streak virus (BSV)* and wheat dwarf virus (WDV) while targeting the conserved genomic elements through CRISPR/SpCas9 [30,32].

CRISPR system comprising the Cas13a and FnCas9 proteins can also target the RNA sequences of RNA viruses. The viral RNA of TuMV was degraded using CRISPR/Cas13a in *N. benthamiana* [91]. Similarly, the CRISPR/FnCas9 system was utilized to target the mutation of “*tobacco mosaic virus (TMV)*” and “*cucumber mosaic virus (CMV*)” in genetically modified *A. thaliana* and *N. benthamiana* [92]. This strategy has also been used to develop resistance against southern rice “*black-streaked draft virus (SRBSDV)*”*,* “*potato virus Y (PVY*)”, and “*rice stripe mosaic virus (RSMV)*”. CRISPR/Cas technology is practical enough to stop the growth of viruses and effectiveness in genetically modified crops [93]. However, there are chances that the virus escapes the system leading to viral infection especially common in the rapidly evolving strains. According to recent research, around 33–48% of viral genomes edited via CRISPR/Cas9 evolved a single nucleotide conserved mutation that was responsible for the resistance, thus resulting in a failed mechanism in Gemini viruses [39,94].

## 4. CRISPR-Based Crop Breeding

**Figure 2:**
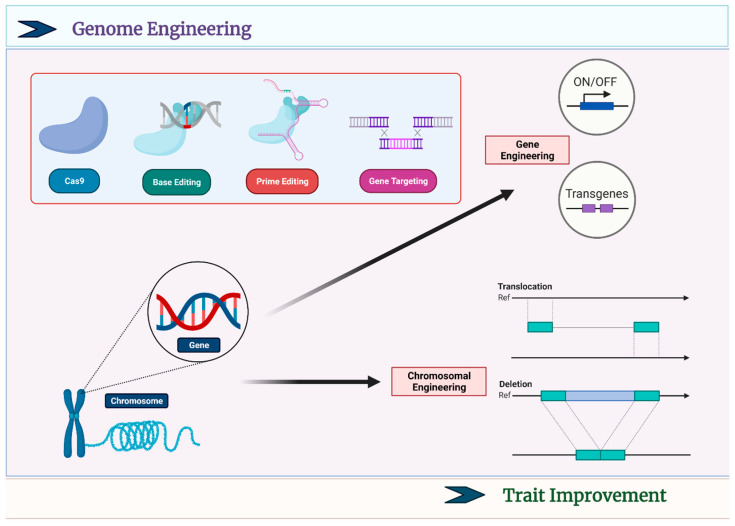
The scheme of Cas9 system-based genome-editing for trait improvement. Cas9 protein is involved in the base editing and prime editing to target genes. Gene engineering in a crop could be achieved by turning ON/OFF a gene by editing its promoter sequence or by introducing a foreign gene (transgene), while chromosome engineering could be achieved by translocation or by creating indels.

CRISPR/Cas system-based gene knock-out technologies can easily engineer resistant plants, as described above. This property of targeted mutagenesis is not only useful for functional genomics but also helpful for crop breeding [95]. A plant variety could be directly and rapidly modified by applying CRISPR technology in crop breeding [96]. The CRISPR/Cas systems were utilized to delete/insert genes in the corn and other important crops, and they showed high performance in the field [97]. The dramatic alterations in phenotype could be achieved by multiple gene knockouts in a crop variety. Figure 2 illustrates the role of genetic engineering in producing plant varieties with improved characteristics. For instance, in-ground cherries and wild tomatoes can create novel crops by knocking out multiple genes for domestication purposes [98,99]. A variety of tomatoes suitable to cultivate in hydroponic cultures have also been developed by inducing multiple gene knockouts by CRISPR/Cas9 system [100]. Besides, knocking out genes the CRISPR technology can also modify the promoter region and upregulate or downregulate the function of desired genes in plants to achieve disease resistance and desirable phenotype [101]. A combination of precise base editing and gene knock-out technologies to modify multiple genes simultaneously is required to improve complex plant traits [102].

## 5. Limitations of CRISPR-Based Tools for Plant Pathogen Resistance

Plant biology has been revolutionized by CRISPR-based technology, and a variety of innovative solutions have been developed to address a variety of challenges [103,104]. The ability to enhance plant resistance to pathogens is one of the most important applications of CRISPR technology [105,106]. Despite recent advances in the field of plant pathogen resistance, there are still several limitations that need to be addressed before CRISPR technology can fully realize its potential in this domain [107,108].

Plant pathogen resistance is limited by the specificity of current CRISPR-based tools [109]. Developing CRISPR-based tools that target specific pathogens is challenging because the specificity of CRISPR is dependent upon the presence of a protospacer adjacent motif (PAM) in the target DNA [110]. Moreover, unintended consequences, such as the disruption of essential genes, cleavage of non-target regions, or the initiation of epigenetic changes, can occur due to off-target effects [111,112]. This can affect the overall health and growth of the plant and reduce the effectiveness of the CRISPR-based tool [113].

The delivery of CRISPR-based plant pathogen resistance tools to target cells is another limitation [114]. The need for efficient and cost-effective methods for delivering CRISPR-based tools to target cells is one of the biggest challenges [115]. Especially in large-scale applications, where delivery costs are a major concern, this may pose a significant hurdle. Additionally, the size of the target DNA limits the efficiency of CRISPR-based tools [116,117]. Designing a CRISPR tool with effective targeting capacity and achieving the desired outcome is more difficult when the target DNA is large [118]. There can be a limitation in the number of targets that can be effectively targeted, thereby limiting the effectiveness of CRISPR-based technology [119]. The durability of CRISPR-based tools is another limitation. A CRISPR-based tool is not permanent and may need to be reapplied periodically to maintain its effectiveness, in contrast to traditional breeding techniques. Reapplication can be costly, especially in large-scale applications [120,121].

## 6. Future Research Directions

The use of CRISPR-based tools has proven to be effective in improving plant resistance to pathogens. Nevertheless, their applications still have limitations and can be improved. Following is a list of possible future developments in this field as well as areas for further research.

The Development of High-Throughput Delivery Methods: Currently, delivering CRISPR components to plants is a challenging task since it requires efficient delivery techniques. CRISPR-based tools can be used to significantly enhance plant resistance with higher speed and efficacy if high-throughput methods are developed to deliver CRISPR components.

Enhanced Targeting Efficiency: Due to the inefficiency of the current methods for delivering CRISPR components into plants, only a small fraction of plants is actually receiving the components. Through further research and development in this area, it will be possible to deliver CRISPR components in an efficient and effective manner, thereby increasing plant resistance against pathogens.

Minimizing Off-Target Effects: There is a risk of off-target effects with CRISPR-based tools, which can lead to unintended changes in the genome. In order to minimize the occurrence of off-target effects, further research is needed to ensure that the modifications made are precise and intended.

Improved Gene Editing Specificity: At present, CRISPR-based tools do not provide very specific results and sometimes cause changes to the surrounding regions of the targeted DNA. Using CRISPR-based tools for enhancing plant resistance would become more accurate and efficient with the development of more specific methods for gene editing.

Implementation of Non-Model Species: Arabidopsis and rice have been used as model species for most of the current research in this field. CRISPR-based tools need to be applied to other plant species, including those of economic importance.

Prolonged Durability of the Changes: CRISPR-based tools currently do not always provide stable modifications to the genome, and these changes may be lost over time. It is essential to conduct research to develop methods for ensuring long-lasting and sustainable changes to the genome.

Integration with Other Plant Breeding Techniques: Plant resistance to pathogens can be further improved with CRISPR-based techniques combined with mutation breeding and chemical mutagenesis. These integration possibilities need to be explored further.

## 7. Conclusions

The CRISPR/Cas systems are widely used tools for genome editing in host plants, addressing problems in plant–pathogen interactions. These systems allow for the introduction of loss-of-function and gain-of-function mutations to understand pathogen–plant interactions and impairments caused by harmful pathogens. CRISPR-based tools can be used for single and multiple genetic transformations and can also generate high-output mutation libraries to accelerate functional genetic studies for enhancing plant resistance. Targeting prospective players in plant–pathogenic interactions, such as R genes, receptor-like kinases, transcription factors, and transcripts, is recommended to achieve effective CRISPR/Cas tool usage. The SNPs, abundant forms of genetic variation in crop species, play a role in crop resistance and can be quickly modified using CRISPR/Cas9-mediated base substitutions in agronomically important crops. A new base editing-mediated genetic editing method has also been developed, allowing the unnatural propagation of endogenous genes in different plants. The various CRISPR/Cas systems and derivatives offer many new opportunities to explore the field of plant disease resistance. As production strategies and plant disease management systems continue to evolve, it is hoped that CRISPR technologies will play a major role in understanding pathogens–plants interactions and designing sustainable disease-resistant plants.

## Figures and Tables

**Table 1:** The various examples of Cas9 nuclease, their targeted plants, PAM sequences, and organisms.

Sr. No.	Cas Nucleases	Targeted Plants	PAM Sequences 5′-3′	Organisms	References
**1**	SpRY	Rice	NGD and NAN	*Streptococcus pyogenes*	[41]
**2**	SpG	Rice	NGD	*Streptococcus pyogenes*	[41]
**3**	SpCas9	Many plants	NGG	*Streptococcus pyogenes*	[47]
**4**	Cas3d/Cas5d/Cas6d/Cas7d/Cas10d	Rice and Tomato	GTH	*Microcystis aeruginosa*	[48]
**5**	NmeCas9	Rice	NNNNGATT	*Neisseria meningitidis*	[49]
**6**	CjCas9	Various plants	NNNNRYAC	*Campylobacter jejuni*	[50]
**7**	Cas14	-	T-rich PAM sequences, eg. TTTA for dsDNA cleavage, no PAM sequence requirement for ssDNA	Uncultivated archea	[51]
**8**	Cas3	-	No PAM sequence needed	in silico analysis of various prokaryotic genomes	[52]
**9**	ScCas9	Rice	NNG	*Streptococcus canis*	[44]
**10**	LbCpf1 (Cas12a)	Rice and Arabidopsis	TTTN (TTTV) (V = A/G/C)	*Lachnospiraceae bacterium ND2006*	[53]
**11**	SaCas9	Arabidopsis, Rice, and Tobacco	NNGRRT	*Staphylococcus aureus*	[54]
**12**	St1Cas9	Arabidopsis	NNAGAAW	*Streptococcus thermophiles*	[55]
**13**	FnCas12a	Rice and Tobacco	TTN	*Francisella novicida*	[56]
**14**	AsCas12a	Rice	TTTN	*Acidaminococcus* sp. *BV3L6*	[57]
**15**	AacCas12b	Cotton	TTN	*Alicyclobacillus acidiphilus*	[58]
**16**	BhCas12b v4	Arabidopsis	ATTN, TTTN, and GTTN	*Bacillus hisashii*	[59]
**17**	AsCpf1 (Cas12a)	Cotton	TTTV	*Acidaminococcus* sp.	[60]

## Data Availability

Data will be available upon request.
